# Behavioral and Gene Expression Analysis of Stxbp6-Knockout Mice

**DOI:** 10.3390/brainsci11040436

**Published:** 2021-03-29

**Authors:** Cong Liu, Qian Hu, Yan Chen, Lingqian Wu, Xionghao Liu, Desheng Liang

**Affiliations:** Center for Medical Genetics, School of Life Sciences, Central South University, Changsha 410008, China; liucong@sklmg.edu.cn (C.L.); huqian@sklmg.edu.cn (Q.H.); chenyan@sklmg.edu.cn (Y.C.); wulingqian@sklmg.edu.cn (L.W.); liuxionghao@sklmg.edu.cn (X.L.)

**Keywords:** Stxbp6, behavior, RNA-seq, weight

## Abstract

Since the first report that Stxbp6, a brain-enriched protein, regulates the assembly of soluble N-ethylmaleimide-sensitive factor attachment protein receptor (SNARE) complexes, little has been discovered about its functions over the past two decades. To determine the effects of Stxbp6 loss on nervous-system-associated phenotypes and underlying mechanisms, we constructed a global Stxbp6-knockout mouse. We found that Stxbp6-null mice survive normally, with normal behavior, but gained less weight relative to age- and sex-matched wildtype mice. RNA-seq analysis of the cerebral cortex of Stxbp6-null mice relative to wildtype controls identified 126 differentially expressed genes. Of these, 57 were upregulated and 69 were downregulated. Moreover, Kyoto Encyclopedia of Genes and Genomes (KEGG) enrichment analysis showed that the most significant enriched KEGG term was “complement and coagulation cascades”. Our results suggest some potential regulatory pathways of Stxbp6 in the central nervous system, providing a remarkable new resource for understanding Stxbp6 function at the organism level.

## 1. Introduction

Neurological disorders were the second leading cause of death worldwide in 2016, exerting a heavy, growing burden worldwide [1]. Neurological disorders require relatively more attention and research support. Various studies suggest that soluble N-ethylmaleimide-sensitive factor attachment protein receptor (SNARE) complexes are involved in neurological diseases [2]. The SNARE complex is a parallel four-helical bundle comprising synaptobrevin-2 (VAMP-2), syntaxin-1, and SNAP-25 [3]. SNARE proteins are essential for membrane fusion and exocytosis [4]. Past studies have shown that VAMP-2-null mice die immediately after birth [5], but VAMP-2-heterozygous-null mice exhibit improved motor coordination [6]. Syntaxin-1 deletion causes loss of neurons and embryonic lethality in mice [7]. Mice with brain-specific SNAP-25 deletion show schizophrenia-like characteristics like hyperactivity, enhanced stereotypical movements, and reduced prepulse inhibition [8].

Amisyn (STXBP6), a small SNARE protein that is a negative regulator of SNARE complex formation, has come to our attention. STXBP6 was first reported to be a brain-enriched protein with a tomosyn- and VAMP-like coiled-coil-forming domain that specifically binds to syntaxin-1 to regulate SNARE complex formation [9]. Later, STXBP6 was found to negatively regulate exocytosis by interfering with the fusion pore [10]. STXBP6 haploinsufficiency was observed in an autistic boy [11]. Stxbp6 gene silencing increased the secretion of large dense-core vesicles (LDCVs) in mouse β-TC3 cells, while its overexpression suppressed secretion [12]. A recent study showed that amysin upregulation suppresses insulin secretion in human β-cells [13]. However, little is known about how Stxbp6 depletion affects nervous-system-associated phenotypes.

Here, we constructed a global Stxbp6-knockout mouse using CRISPR/Cas9 and carried out behavioral and gene expression analysis. We found that Stxbp6-null mice survive normally but gain less weight than age- and sex-matched wildtype mice. Stxbp6-null mice exhibited changes in transcriptomes involving a series of genes, including Il22 and C3. Together, these findings suggest that Stxbp6 has important biological functions, though its depletion may be insufficient to induce severe neurological disorders, at least in our experimental setting.

## 2. Materials and Methods

### 2.1. Mouse Model

Mice used in this study had a C57BL/6 genetic background and were maintained under 12/12 h light/dark cycles with ad libitum access to food and water. Stxbp6-knockout mice were created using CRISPR/Cas9 to delete exon 3 and flanking sequences (total size: 707 bp) on Stxbp6 (Cyagen Biosciences, Suzhou, China). Mice were killed by cervical dislocation, and tissues were isolated by handpicking. The study protocol was approved by the School of Life Sciences of Central South University Institutional Animal Care and Use Committee (2015031304).

### 2.2. Genotyping

Genomic DNA was extracted from mouse tail tissue using the Trelief Animal Genomic DNA Kit (TsingKe Biotech, Beijing, China). Genotypes were determined by PCR using 2× Phanta Max Master Mix (Vazyme, Nanjing, China) using the following primers: 5′-gcatcgcagccagtggtgtt-3′ (forward) and 5′-gggcattaggaggcaaatgaaatt-3′ (reverse). DNA electrophoresis was done on 1% agarose gels with wildtypes and homozygotes producing single bands of 1306 and 599 bp, respectively. Heterozygotes produced both bands.

### 2.3. Western Blot Analysis

Protein was extracted from the brain tissues, denatured in 2× SDS sample loading buffer (Beyotime Biotechnology, Shanghai, China) for 10 min at 95 °C, and then resolved on 12% SDS-PAGE (sodium dodecyl sulfate–polyacrylamide gel electrophoresis). Proteins were then transferred onto PVDF (polyvinylidene fluoride) membranes and blocked with 5% nonfat milk for 1 h, at room temperature. They were then incubated in primary antibodies against Stxbp6 (antibodies-online, Atlanta, GA, USA) and β-actin (Cell Signaling Technology, Danvers, MA, USA) overnight at 4 °C. Membranes were washed thrice with 1× PBST and incubated in anti-rabbit IgG (Jackson ImmunoResearch, West Grove, PA, USA) secondary antibody for 1 h, at room temperature. After washing, a chemiluminescent signal was developed and imaged on X-ray film on a ChemiDoc XRS+ system (Bio-Rad, Hercules, CA, USA).

### 2.4. Behavioral Analysis

The open field test, rotor-rod test, three-chamber test, and Morris water maze (MWM) were carried out as previously described [14,15], with modifications. Briefly, in open field tests, mice were placed in an open field chamber (an opaque plastic box: length 72 cm, width 72 cm, and height 40 cm) for 10 min. The total distance moved, traveled distance, and time spent in the central area (36 × 36 cm) were recorded using video tracking software (Anilab, Ningbo, China). In rotor-rod tests, mice were placed on the rotating rod (20 rpm); automatic sensors were used to capture when mice fell off the rod and the latency to fall was recorded. The three-chamber test was done in a three-chambered box (length 20 cm, width 40 cm, height 30 cm) with openings between the chambers, with the left and right chambers separated by the middle chamber containing a wire cage. First, two wire cages were empty at Stage 1. Then, a never-before-met mouse (Stranger 1) was placed under the left wire cage at Stage 2. Finally, the second never-before-met mouse (Stranger 2) was placed under the right wire cage at Stage 3. The experimental mouse was placed in the middle chamber for 10 min in each stage, and time spent with the empty wire cage or another mouse was recorded. In the MWM test, mice were placed in a MWM circular water tank, containing water opacified with milk and a hidden platform placed about 1 cm below the water surface. At the pre-training stage, the mouse was placed into the water at one of four starting quadrants and trained to find the platform within 60 s. At the post-training stage, the platform in the target quadrant was removed from the water tank, and mice were allowed to swim for 60 s. Time spent in the target quadrant, the latency in finding the position where the platform had been in the pre-training stage, and the number of times the mouse crossed were recorded. Body weight was taken on an electronic balance.

### 2.5. Differential Expression (DE) Analysis

The R (v3.5.3) package [16] DESeq2 (v1.22.2) [17] was used to identify differentially expressed genes (DEGs) between two groups using the raw counts table with raw reads mapped to each gene (row), for each sample (column) as input. Statistically significant DEGs were identified using |log_2_ FoldChange| ≥ 1 and *p* < 0.05 as cutoffs.

### 2.6. Enrichment Analysis

To assess the functions of DEGs, Gene Ontology (GO) and Kyoto Encyclopedia of Genes and Genomes (KEGG) enrichment analyses of the DEGs were performed using the enrichGO and enrichKEGG functions of the clusterProfiler (v3.10.1) package [18] in R, respectively. The significant GO terms were annotated and plotted using the dotplot function of clusterProfiler package, as were the KEGG pathways. Benjamini–Hochberg-adjusted *p* < 0.05 indicated statistical significance.

### 2.7. Protein–Protein Interaction (PPI) Network Analysis

A PPI network of DEGs was constructed and visualized using the Search Tool for the Retrieval of Interacting Genes (STRING) database (v11.0) [19] and Cytoscape (v3.6.1) [20] software, respectively. In the data settings on the STRING website, we changed the parameters as follows: (1) meaning of network edges: evidence; (2) active interaction sources: Textmining & Experiments & Databases & Co-expression; (3) minimum required interaction score: highest confidence (0.900); and (4) display simplifications: hide disconnected nodes in the network. Crucial genes in the PPI network were identified using the cytoHubba (v0.1) [21] plugin on Cytoscape, using the maximal clique centrality (MCC) method to identify important subnetworks and hub genes.

### 2.8. Quantitative Real-Time Polymerase Chain Reaction (QRT-PCR)

Gene expression analysis was done using species-specific QRT-PCR. Primers were designed using NCBI’s Primer-BLAST [22]. Primer sequences are shown in Table S3. RNA was extracted from tissues and cells using TRIzol Reagent (Invitrogen, Carlsbad, CA, USA) and reverse-transcribed using HiScript II Q RT SuperMix with gDNA wiper (Vazyme, China) following the manufacturer’s instructions. QRT-PCR was performed on a CFX96 Touch Real-Time PCR system (Bio-Rad, USA), using ChamQ Universal SYBR qPCR Master Mix (Vazyme, China). The QRT-PCR protocol was as follows: 1 cycle of 30 s at 95 °C, followed by 40 cycles of 10 s at 95 °C and 30 s at 60 °C. Samples were analyzed in triplicate, and the relative gene expression was determined using the 2^−ΔΔCt^ method using beta-actin as a reference gene [23]. Statistical significance between two groups was determined via Student’s *t*-test on GraphPad Prism (v6.01). *p* < 0.05 indicated statistical significance.

## 3. Results

### 3.1. Global Stxbp6-Knockout Mouse Model

**Figure 1:**
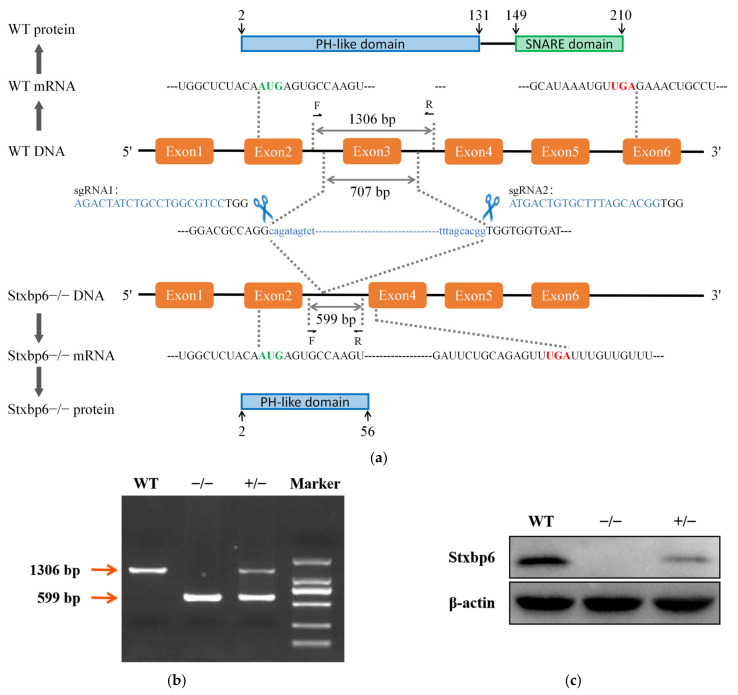
CRISPR/Cas9-mediated generation of Stxbp6-knockout mice. (**a**) Schematic diagram of the CRISPR/Cas9 targeting sites on Stxbp6; (**b**) PCR genotyping of Stxbp6 wildtype (WT) (1306 bp), heterozygous (1306 bp and 599 bp), and homozygous (599 bp) mice. (**c**) Stxbp6 protein expression analysis by Western blot. β-actin was used as a loading control.

To establish the global Stxbp6-knockout mouse, we designed two sgRNAs targeting exon 3 on Stxbp6 so as to completely excise exon 3 and cause premature termination of translation in exon 4. The protein that might be generated after the removal of exon 3 only retained part of the pleckstrin homology (PH-like) domain (Figure 1a). Deletion of the 707 bp target sequence was verified by Sanger sequencing (Figure S2). No off-target sites were observed in the initial three mice (Tables S1 and S2 and Figure S1). Mouse genotypes were confirmed by PCR (Figure 1b), and Stxbp6 protein levels were determined by Western blot analysis (Figure 1c). In wildtype (WT) mice, a 24 kDa band was evident, but this band was absent and reduced in Stxbp6-knockout and heterozygous mice, respectively. After months of breeding and observation, we found that Stxbp6-null mice are viable. These Stxbp6-null mice are a valuable tool for investigating Stxbp6 in vivo.

### 3.2. No Obvious Behavioral Abnormalities Were Observed in Stxbp6-Knockout Mice

**Figure 2:**
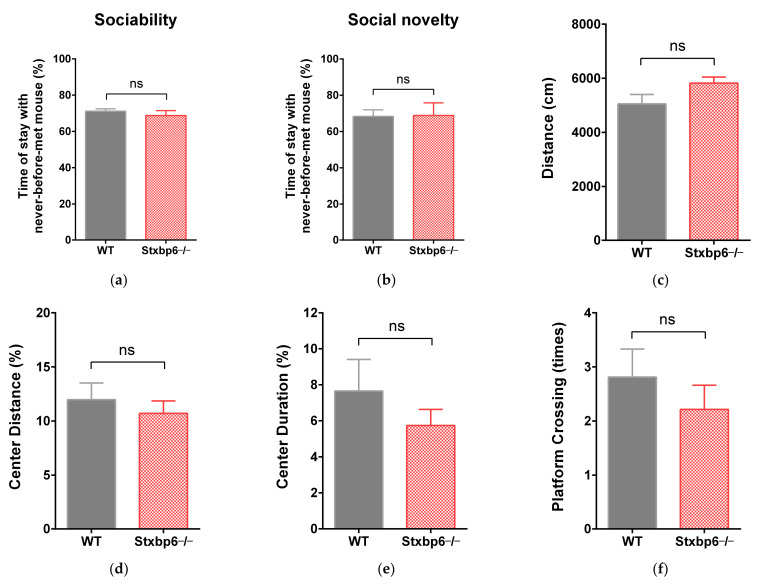

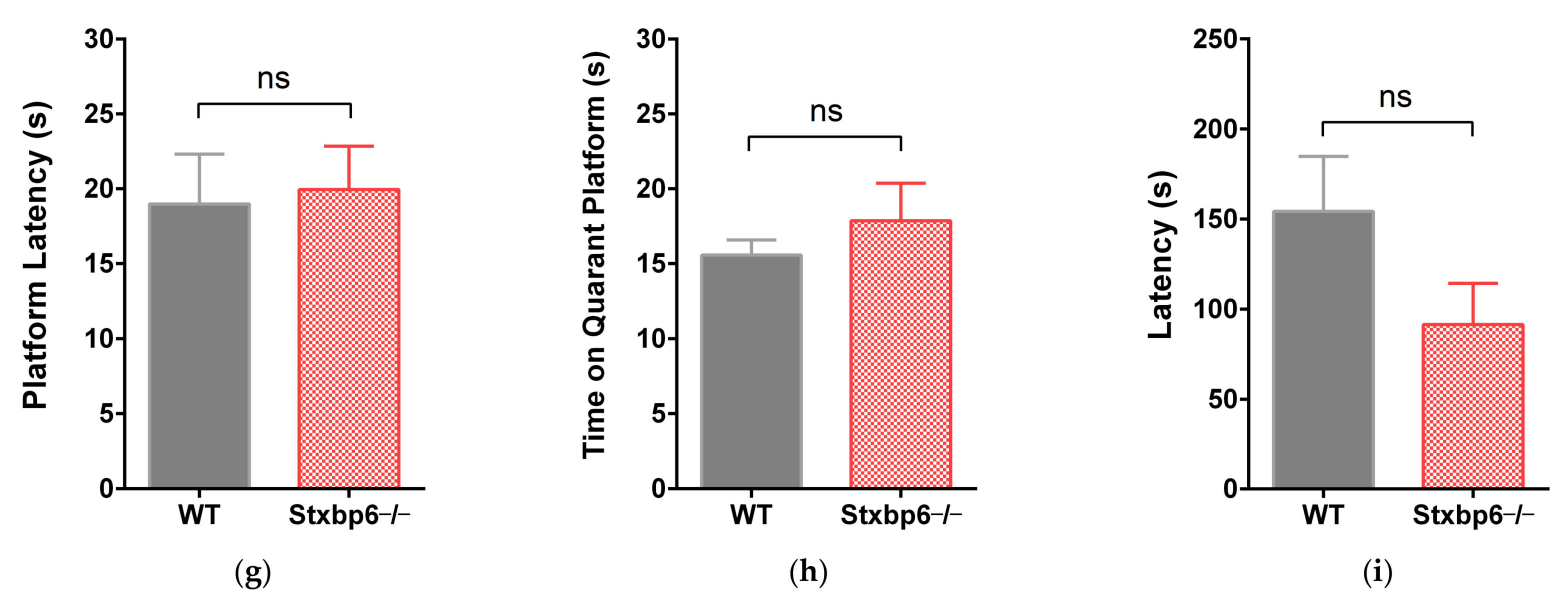
Behavioral analyses of Stxbp6-knockout mice. (**a**) Time (%) with the new stranger spent by Stxbp6−/− mice was not significantly different compared to that by WT mice (WT: 71.15 ± 1.307, Stxbp6−/−: 68.75 ± 2.726; *p* = 0.4427) in the sociability stage of the three-chamber test (**b**) or in the social novelty stage (WT: 68.35 ± 3.631, Stxbp6−/−: 68.83 ± 6.994; *p* = 0.9522); (**c**) The open field test was used to evaluate the general activity levels of the mice. No difference was observed in total distance moved (WT: 5052 ± 348.3, Stxbp6−/−: 5819 ± 228.0; *p* = 0.0743), (**d**) percentage of distance spent in the central area (WT: 11.98 ± 1.533, Stxbp6−/−: 10.71 ± 1.149; *p* = 0.5116), (**e**) or percentage of time spent in the central area (WT: 7.656 ± 1.750, Stxbp6−/−: 5.740 ± 0.8922; *p* = 0.3287) between WT and Stxbp6−/− mice; (**f**) The following parameters in the Morris water maze were assessed: number of platform crossings in the target quadrant (WT: 2.813 ± 0.517, Stxbp6−/−: 2.2140 ± 0.448; *p* = 0.4045), (**g**) the latency in finding the platform (WT: 18.99 ± 3.326, Stxbp6−/−: 19.96 ± 2.893; *p* = 0.8317), (**h**) and time spent in the target quadrant (WT: 15.58 ± 1.012, Stxbp6−/−: 17.86 ± 2.519; *p* = 0.3930); (**i**) Latency to fall was recorded in constant-speed mode in the rotor-rod test, and no significant difference was found between WT and Stxbp6−/− mice (WT: 154.2 ± 30.58, Stxbp6−/−: 91.40 ± 22.98; *p* = 0.1145). Note: Data are shown as mean ± SEM, n ≥ 7 per group. Unpaired two-tailed Student’s *t*-test was used. ns, not significant.

STXBP6 is reported to be a brain-enriched SNARE protein and has been considered a candidate gene for autism spectrum disorder (ASD). Next, we performed a series of behavioral tests on Stxbp6-null mice to assess whether they have neurological disorder phenotypes. In the three-chamber test, after habituation to the empty box, time with the new stranger spent by Stxbp6−/− mice was not significantly different compared to that by WT mice both in the sociability stage (Figure 2a) and the social novelty stage (Figure 2b). Open field tests to assess the general activity levels of the mice showed that the total distance moved, percentage of distance spent in the central area, and percentage of time spent in the central area did not differ significantly in Stxbp6−/− versus WT mice (Figure 2c–e). The MWM and rotor-rod tests to evaluate spatial memory and sensorimotor coordination, respectively, revealed that Stxbp6−/− mice did not display motor impairments in the rotor-rod test (Figure 2i), and their spatial memory was similar to that of WT mice in the MWM (Figure 2f–h). Together, these findings suggest that Stxbp6 deletion did not affect mouse sensorimotor, learning, memory, or social interaction capacity.

### 3.3. Comparison of Gene Expression Profiles between Cerebral Cortexes from WT and Stxbp6−/− Mice

**Figure 3:**
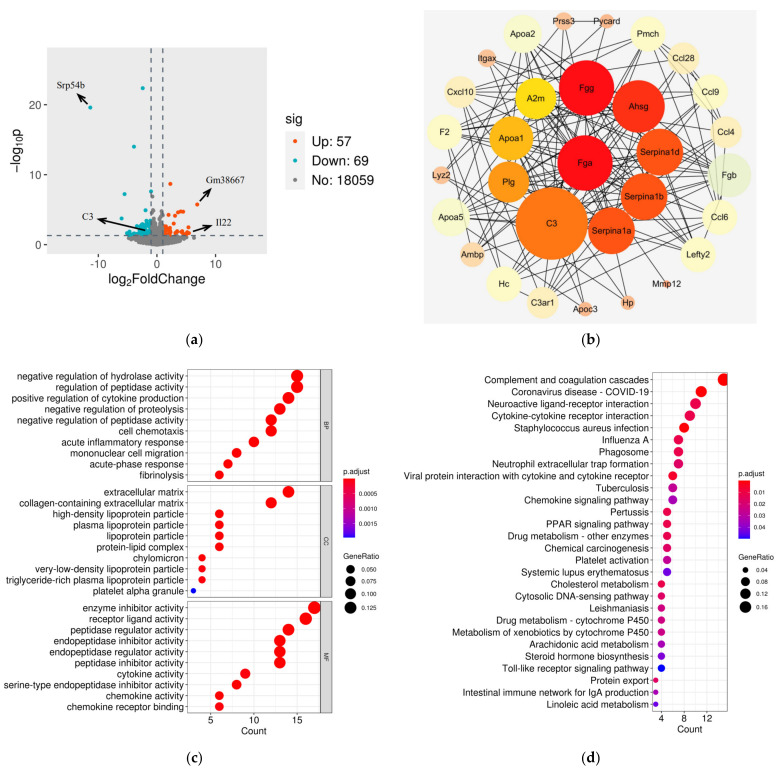
Comparison of mRNA expression profiles between two groups. (**a**) Volcano plot of mRNA expression data. Blue and red dots represent differentially expressed genes (DEGs) filtered based on cutoff values |log_2_ FoldChange| ≥ 1 and *p* < 0.05. Grey dots represent genes with no significant differential expression; (**b**) Protein–protein interaction (PPI) network of DEGs. Circles represent genes and black lines represent interaction partners of the DEGs. Each circle is color-coded according to score using the maximal clique centrality (MCC) method. The larger red and orange circles show the hub genes. (**c**) Dot plot of the most significantly enriched Gene Ontology (GO) (BP: biological process, CC: cellular component, MF: molecular function) terms. Each dot is color-coded according to the adjusted *p* value. Dot size corresponds to gene count; (**d**) Dot plot of all significantly enriched Kyoto Encyclopedia of Genes and Genomes (KEGG) pathways.

To investigate the impact of Stxbp6 knockout on the brain, we carried out transcriptome analysis on the cortexes of Stxbp6-null (n = 3, KO) and wildtype mice (n = 3, WT) and identified 126 differentially expressed genes (DEGs) in the KO group, of which 57 were upregulated and 69 were downregulated (Figure 3a and Figure S4 and Table S4). Of the DEGs, Il22 was the most differentially expressed (log_2_ FoldChange = 5.45, *p* < 0.05), while Gm38667 was a predicted gene. Srp54b was most downregulated (log_2_ FoldChange = -11.36852577, *p* < 0.05). Srp54a and Srp54c were also downregulated. PPI analysis of the DEGs identified the top 10 genes as C3, Fga, Fgg, Ahsg, Serpina1d, Serpina1b, Serpina1a, Plg, Apoa1, and A2m, as ranked by the MCC method (Table S5), and that C3 was centrally located in the PPI network (Figure 3b). Validation by QRT-PCR confirmed the expressional trends of C3 and Il22 mRNA, as well as the three Srp54 genes, observed in the RNA-seq analysis (Figure S3).

To further define the functionality of the DEGs, we carried out GO and KEGG enrichment analysis. GO analysis found the DEGs to be significantly enriched in 464 GO terms, with 399 related to Biology Process (BP), 14 related to Cellular Component (CC), and 51 related to Molecular Function (MF). Moreover, GO analysis showed that BP was mainly enriched in “negative regulation of hydrolase activity”, “regulation of peptidase activity”, “positive regulation of cytokine production”, “negative regulation of proteolysis”, and “negative regulation of peptidase activity”. The mainly enriched terms in CC were “extracellular matrix”, “collagen-containing extracellular matrix”, “high-density lipoprotein particle”, “plasma lipoprotein particle”, and “lipoprotein particle”. The most significantly MF terms were “enzyme inhibitor activity”, “receptor ligand activity”, “peptidase regulator activity”, “endopeptidase inhibitor activity”, and “endopeptidase regulator activity” (Figure 3c, Table S6). KEGG analysis identified 28 significantly enriched KEGG pathways, mainly including “Complement and coagulation cascades”, “Coronavirus disease—COVID-19”, “Staphylococcus aureus infection”, “Viral protein interaction with cytokine and cytokine receptor”, and “Pertussis” (Figure 3d, Table S7). These results imply that Stxbp6 knockout has far-reaching effects.

### 3.4. Stxbp6-Knockout Mice Were Leaner Than Age- and Sex-Matched Wildtype Mice

**Figure 4:**
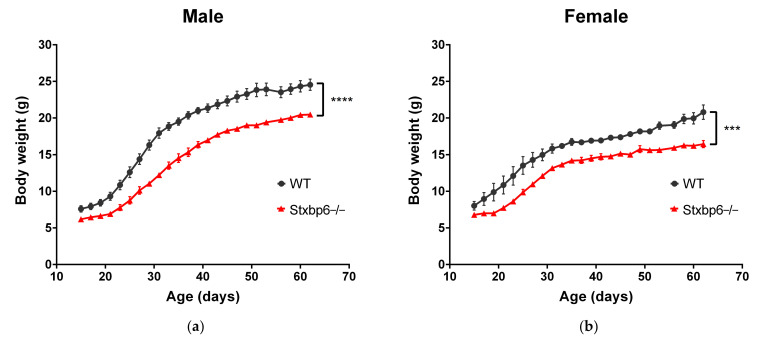
Mouse weights. (**a**) Monitoring of body weight over 7 weeks in male mice. Body weight effects: genotype (F(1, 11) = 35.76, *p* < 0.0001), age (F(23, 253) = 660.9, *p* < 0.0001); (**b**) Monitoring of body weight over 7 weeks in female mice. Body weight effects: genotype (F(1, 9) = 30.64, *p* = 0.0004), age (F(23, 207) = 141.4, *p* < 0.0001). *** *p* < 0.001; **** *p* < 0.0001.

With the same chow feeding, we found that male (Figure 4a) and female (Figure 4b) Stxbp6−/− mice gained less body weight relative to age-matched WT controls from 2 to 9 weeks of age (two-way ANOVA: *p* < 0.0001, and *p* = 0.0004, respectively). On average, at 9 weeks, male and female Stxbp6−/− mice gained 4.065 (95% CI = 1.619–6.512, *p* < 0.0001) and 4.358 (95% CI = 1.978–6.737, *p* < 0.0001) grams less, respectively, relative to WT mice.

## 4. Discussion

Stxbp6 is a small, brain-enriched SNARE protein that can negatively regulate SNARE complex formation. Some studies have indicated that Stxbp6 has important roles and is involved in the occurrence and development of many diseases, including neurological disorders, diabetes, and cancer [24]. However, it is not clear whether loss of Stxbp6 causes abnormal nervous-system-associated phenotypes in mice. Due to the high degree of genetic and physiologic homology between rodents and humans, mice have been the preferred animal models for biomedical research in the last several decades [25,26]. Although transgenic animal models have inherent limitations and need improvement, we have understood many pathologies and molecular mechanisms of human nervous system disorders through different animal models [27,28]. It is essential to develop Stxbp6 mouse models. Here, we developed an Stxbp6-deficient mouse model and conducted behavioral and gene expression analysis.

Although Stxbp6 is brain-enriched, unlike deletions of other SNARE proteins such as VAMP2, SNAP-25, and syntaxin-1, Stxbp6 deletion was not fatal and had mild phenotypes. The performance of Stxbp6−/− mice in the three-chamber test, open field test, Morris water maze test, and rotor-rod test was similar to that of wildtype controls, suggesting that Stxbp6 deletion has limited influence on mouse social, motor, learning, and spatial memory ability. In addition to the possibility that there are no abnormal phenotypes in Stxbp6−/− mice, they may not be found under the experimental conditions we designed, or there are other conditions that we have not tested [29].

Transcriptome analysis of Stxbp6−/− cerebral cortexes relative to wildtype controls identified 126 DEGs, of which Il22 was the most upregulated. Il22 upregulation has also been reported in the BTBR mouse model of autism [30]. Additionally, PPI analysis, GO, and KEGG analysis found that the complement protein C3 and “complement and coagulation cascades” were most outstanding. C3, a central component in the complement and coagulation cascades signaling pathway, was downregulated in our study. Further, mice with C3 knockdown in the prefrontal cortex exhibit social interaction deficits and repetitive behavior [31]. Activated complement and coagulation cascades present in lung adenocarcinoma [32], cholangiocarcinoma [33], and breast cancer [34] patients. However, having said that, the paucity of data on the protein expression of cytokines and molecules related to neuroinflammation did not allow us to conclude at this stage that depletion of Stxbp6 may trigger an immune-related response in the cerebral cortex of mice.

Interestingly, we found that Stxbp6 depletion led to a reduction in body weight. Some ASD-related mice present loss in body weight. C57BL/6J mice carrying 3q29 deletion show slightly reduced body weight and have typical ASD-related behavioral phenotypes [35]. Densin-knockout mice also show mental illness behavior and low body weight [36]. However, a change in body weight can also be due to metabolic changes. Down-regulation of Stxbp6 can stimulate glucose-induced growth hormone release in INS-1 832/13 rat insulinoma cells. Overexpression of Stxbp6 can suppress hormone release in INS-1 832/13 rat insulinoma cells and human β-cell line EndoC-βH2 [13]. Considering that Stxbp6 can regulate exocytosis [9,10,12], a primary weakness in the present study is that we did not examine vesicular fusion and release in Stxbp6−/− mice. Thus, we cannot exclude the possibility that the observed differences in weight between Stxbp6-knockout and WT mice were a consequence of abnormal metabolism. Metabolism and/or gastrointestinal function in Stxbp6−/− mice should be a focus of future study.

## 5. Conclusions

Here, we found that global Stxbp6-knockout mice are viable and have normal sensorimotor, learning, memory, and social interaction ability. However, Stxbp6-knockout mice gained less body weight. Our data offer a new perspective on Stxbp6 function. Our Stxbp6-null mouse model lays the foundation for in-depth study of the function and pathogenic mechanism of Stxbp6 and the development of potential treatments.

## Data Availability

Sequencing data have been deposited in GEO under accession code GSE169726.

## Supplementary Materials

The following are available online at https://www.mdpi.com/article/10.3390/brainsci11040436/s1, Table S1: Candidate CRISPR/Cas9 off-target loci, Table S2: CRISPR/Cas9 and primers for off-target sites, Table S3: Primer sequences, Table S4: All differentially expressed genes in the KO group, Table S5: The top 10 genes in the network as ranked using the MCC method, Table S6: GO enrichment analysis, Table S7: KEGG enrichment analysis, Figure S1: Sanger sequencing of the top 10 off-target sites. Figure S2: Sanger sequencing of PCR products, Figure S3: The mRNA expression levels of C3, Il22, and three Srp54 genes in the cortex were verified by QRT-PCR, Figure S4: Quality control of sequencing data, Figure S5: Heatmap to visually assess the results of clustering on the DEG expression profile.
