# Supplementary material for: Behavioral and Gene Expression Analysis of Stxbp6-Knockout Mice
Source: Brain Sci. 2021 Mar 29;11(4):436. doi: 10.3390/brainsci11040436 (PMC8066043; doi:10.3390/brainsci11040436)
Supplement: Supplementary file 1 [file brainsci-11-00436-s001.zip › FigureS1.pdf]

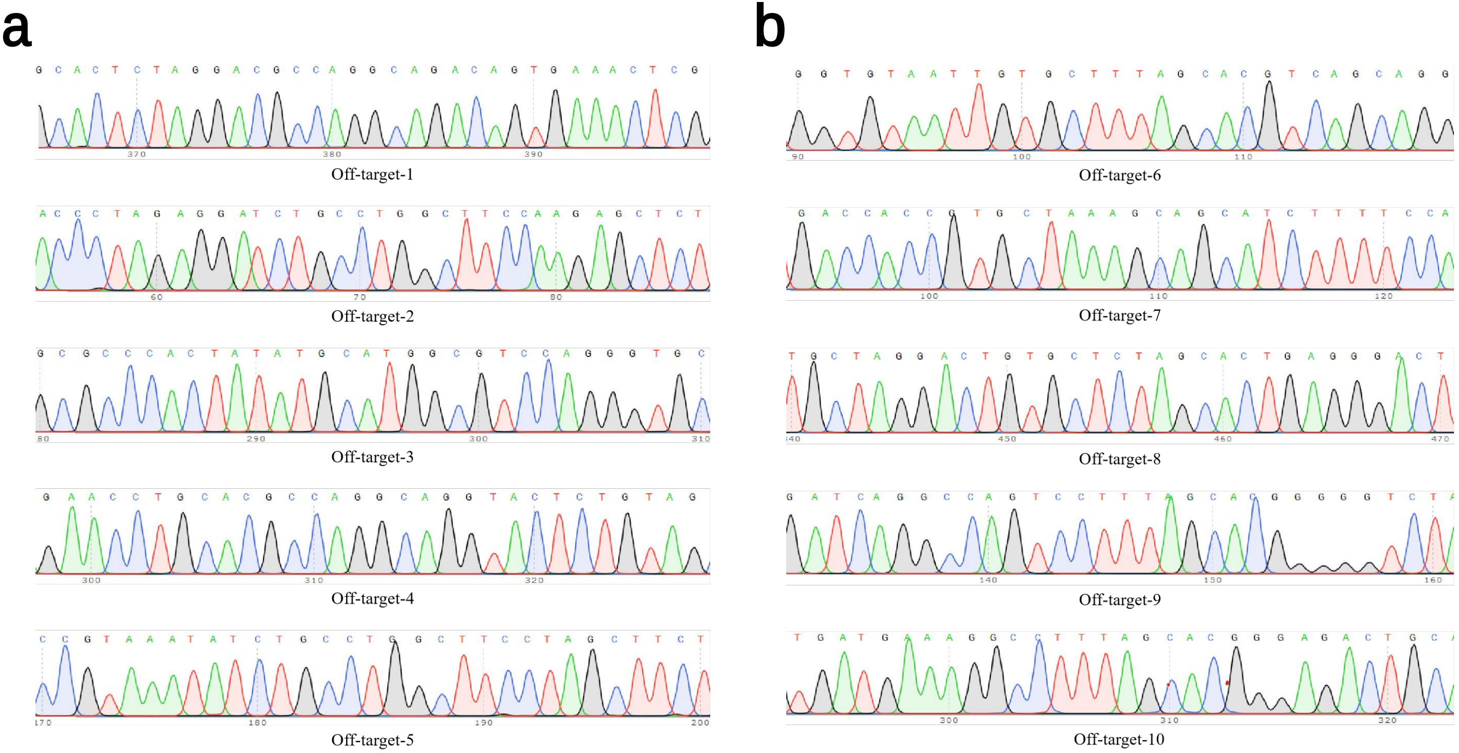
a
b
Off-target-1
Off-target-2
Off-target-3
Off-target-4
Off-target-5
Off-target-6
Off-target-7
Off-target-8
Off-target-9
Off-target-10
