# Supplementary material for: Behavioral and Gene Expression Analysis of Stxbp6-Knockout Mice
Source: Brain Sci. 2021 Mar 29;11(4):436. doi: 10.3390/brainsci11040436 (PMC8066043; doi:10.3390/brainsci11040436)
Supplement: Supplementary file 1 [file brainsci-11-00436-s001.zip › brainsci-1122874_supplementary.docx]

| **Off-target site** | **Sequence** | **Mismatches** | **Locus** |
| --- | --- | --- | --- |
| Off-target-1 | TCACTGTCTGCCTGGCGTCCTAG | 3 | Chr4:100493358 |
| Off-target-2 | AGAGGATCTGCCTGGCTTCCAAG | 3 | Chr7:142887421 |
| Off-target-3 | CCACTATATGCATGGCGTCCAGG | 4 | Chr2:164990527 |
| Off-target-4 | AGAGTACCTGCCTGGCGTGCAGG | 3 | Chr11:107308279 |
| Off-target-5 | TAAATATCTGCCTGGCTTCCTAG | 4 | Chr10:116082223 |
| Off-target-6 | GTAATTGTGCTTTAGCACGTCAG | 4 | Chr1:156564095 |
| Off-target-7 | AAGATGCTGCTTTAGCACGGTGG | 4 | Chr19:55507612 |
| Off-target-8 | AGGACTGTGCTCTAGCACTGAGG | 3 | Chr18:79047113 |
| Off-target-9 | AGGCCAGTCCTTTAGCACGGGGG | 4 | Chr13:93377573 |
| Off-target-10 | ATGAAAGGCCTTTAGCACGGGAG | 4 | Chr7:138189972 |

**Table S1.** The candidate off-target loci of CRISPR/Cas9 in the genome.

**Table S2.** CRISPR/Cas9 and primers used in the study.

| **Name** | **Sequence (5’-3’)** |
| --- | --- |
| gRNA1 | AGACTATCTGCCTGGCGTCCTGG |
| gRNA2 | ATGACTGTGCTTTAGCACGGTGG |
| Off-target-1 | Forward: TCATCTTCGTCCAGCCTCAC |
|  | Reverse: CGGGCATTTCAACTCCTTTT |
| Off-target-2 | Forward: ACAAGGAAGCAATCTGTAAAACG |
|  | Reverse: TGTCCTTGGTTGGTTCCTCC |
| Off-target-3 | Forward: GACCCAACTCCACTTCTCCA |
|  | Reverse: CAGCAGGCATCACTAATCTTT |
| Off-target-4 | Forward: TAACGGTTGACATCTTGGAG |
|  | Reverse: CGGAAATAGGCATAGCACAG |
| Off-target-5 | Forward: ATTCTGCCAGGATCTTTCACC |
|  | Reverse: AGCGGGTCTCCCTTGTAGTTT |
| Off-target-6 | Forward: GCTTGTTAAATTATCCCAAGTTCTC |
|  | Reverse: CTGACAGCCCTGGATGAGATGC |
| Off-target-7 | Forward: ACTTCCTTTGCCTGTTCCTTGA |
|  | Reverse: GTGGTGGTCTTACCTGTGCTTG |
| Off-target-8 | Forward: ATATCATATCGCCTTCCTCCCA |
|  | Reverse: CCCAAGTTTGCTTTGCACCAT |
| Off-target-9 | Forward: GACGGGAAGATACCTCCAAAT |
|  | Reverse: GCACTTGCCTAGCCTACCG |
| Off-target-10 | Forward: CACCACCCACCTGAGAAT |
|  | Reverse: CCGCAGGGATACAACATAG |

| 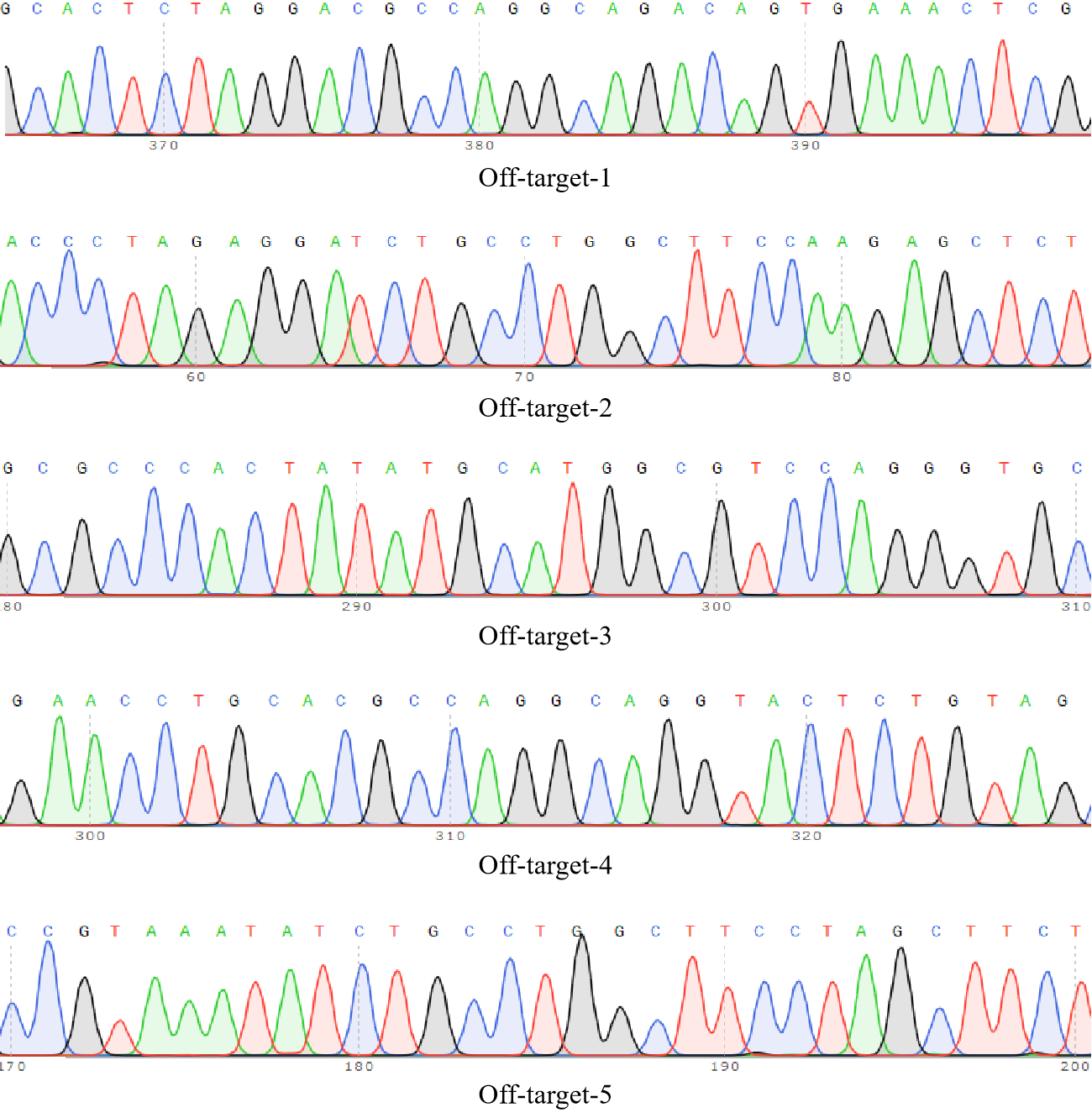 | 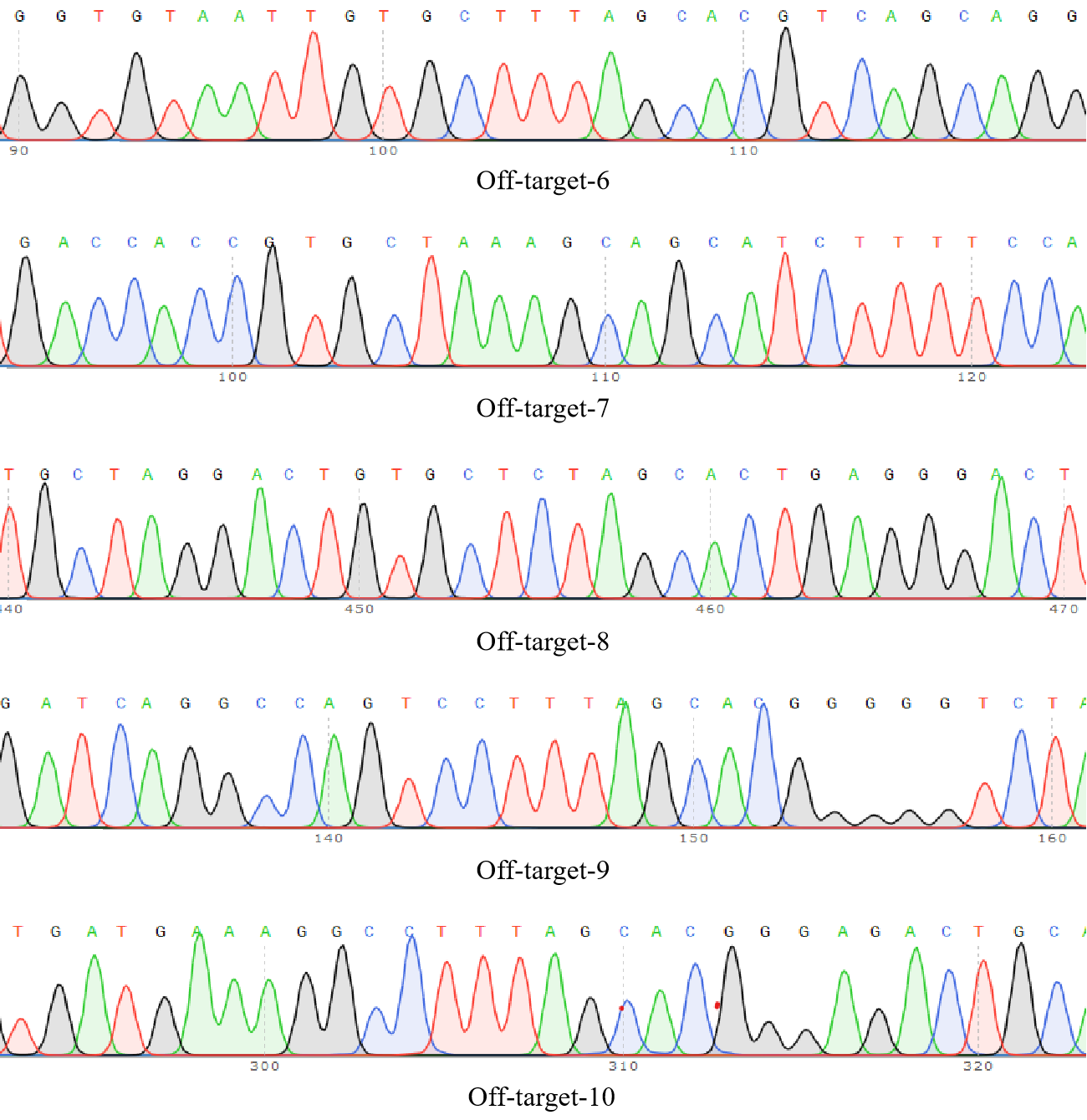 |
| --- | --- |
| (**a**) | (**b**) |

**Figure S1.** Sanger sequencing of the top10 off-target sites. We analyzed the genome DNA of the initial three mice. No indels at the sites were found.

| 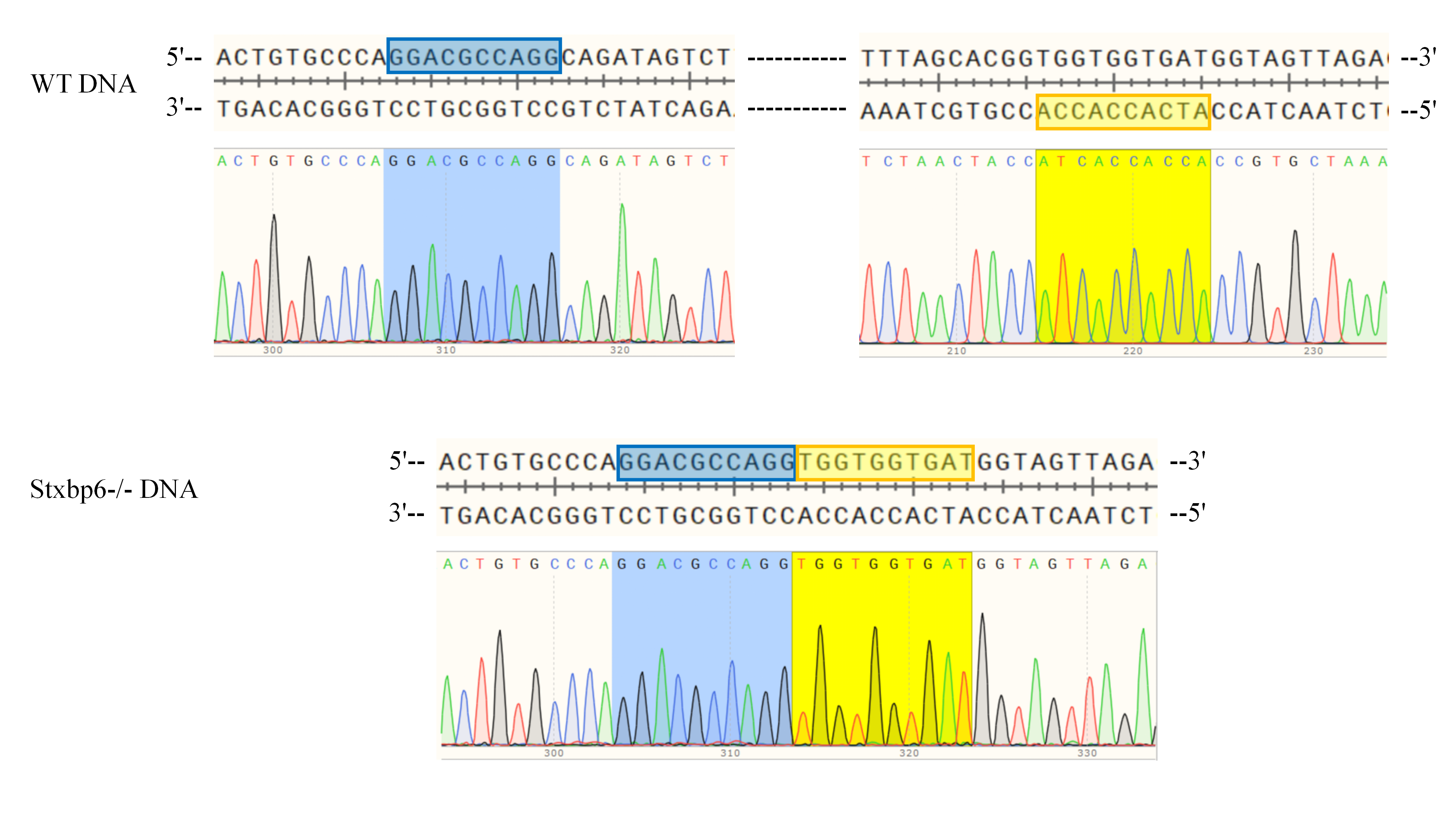 |
| --- |

**Figure S2.** The PCR products were sequenced by Sanger sequencing.

**Table S3.** Primer sequences.

| **Gene symbol** | **GenBank Accession** | **Primers (5’-3’)** | **Product length (bp)** |
| --- | --- | --- | --- |
| Beta-actin | NM_007393.5 | Forward: CACTGTCGAGTCGCGTCC | 89 |
|  |  | Reverse: TCATCCATGGCGAACTGGTG |  |
| C3 | NM_009778.3 | Forward: ACCCCTTCATTCCTTCCACC | 130 |
|  |  | Reverse: GAGTAATGATGGAATACATGGGGA |  |
| Il22 | NM_016971.2 | Forward: ATCAGCTCAGCTCCTGTCAC | 123 |
|  |  | Reverse: CCAGTTCCCCAATCGCCTTG |  |

| 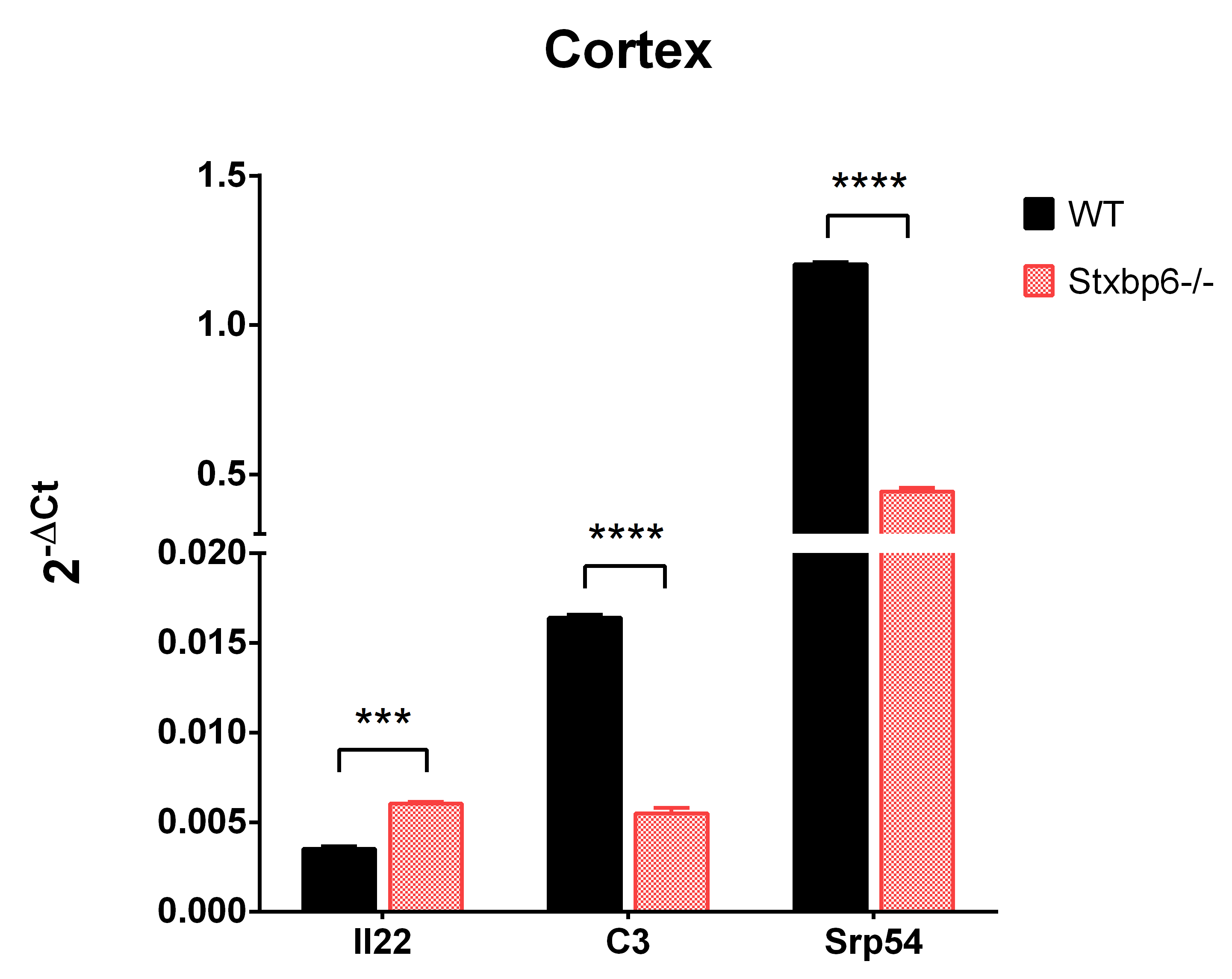 |
| --- |

**Figure S3.** The mRNA expression level of Il22, C3 and Srp54 (Srp54a, Srp54b, and Srp54c) in the cortex was verified by QRT-PCR. Data are expressed as mean±SEM of 3 independent experiments. Unpaired two-tailed student’s *t*-test. * *P* < 0.05, ** *P* < 0.01, *** *P* < 0.001, **** *P* < 0.0001, ns means not significant.

**Sequencing and Data Preprocessing**

Stxbp6-/- (n=3, KO) and wildtype (n=3, WT) mice cortex were used for whole transcriptome sequencing analysis. RNA-seq was performed on the DNBSEQ platform by MGI Technology Co., Ltd, Guangdong, China. Sequencing data filtering were processed using SOAPnuke (v1.5.2) software [1]. Three major preprocessing steps that clean up the raw sequencing reads are: (1) removing reads with adaptors; (2) removing low-quality reads (percentage of bases which quality is lesser than 10 is more than 20% in a read); (3) removing reads with more than 5% of unknown bases and very short reads (less than 20 bases in a read). The reads quality metrics were assessed based on Q30, the rate of bases which quality is greater than 30. After cleaning up, clean reads were aligned to the Mus musculus reference gene sequence (transcriptome) using Bowtie2 (v2.2.5) software [2] followed by mRNA expression level determination using RSEM (v1.2.8) software [3]. Finally, the average number of raw reads and clean reads obtained by sequencing the samples are 23.31M and 23.23M, with a minimum of 22.06M and 22.01M, respectively (Figure S4a). Both Q20 and Q30 of each samples were larger than 92%, with a minimum of 97.15% and 92.49%, respectively (Figure S4b). Each sample was well sequenced, and we used it for downstream analysis.

| 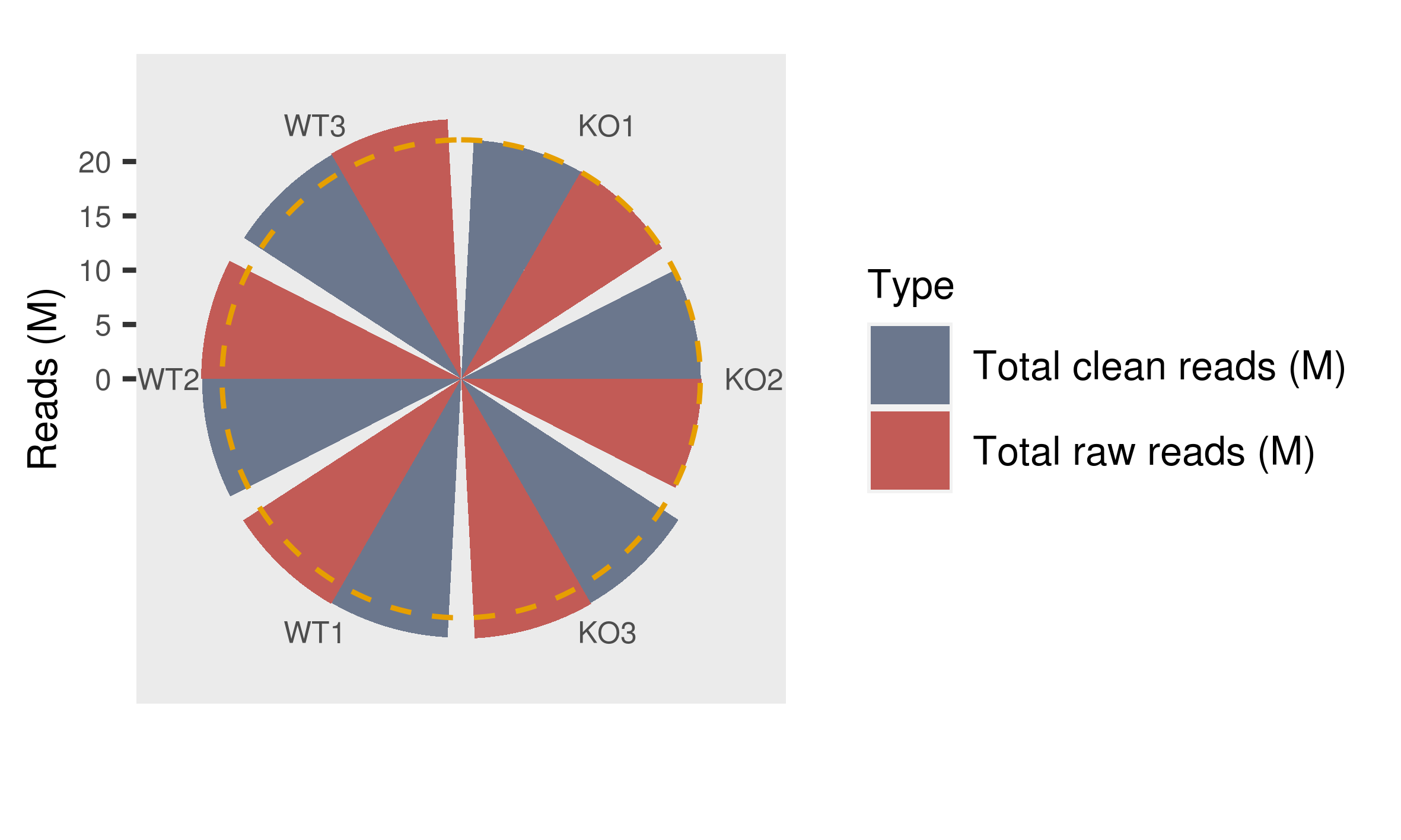 | 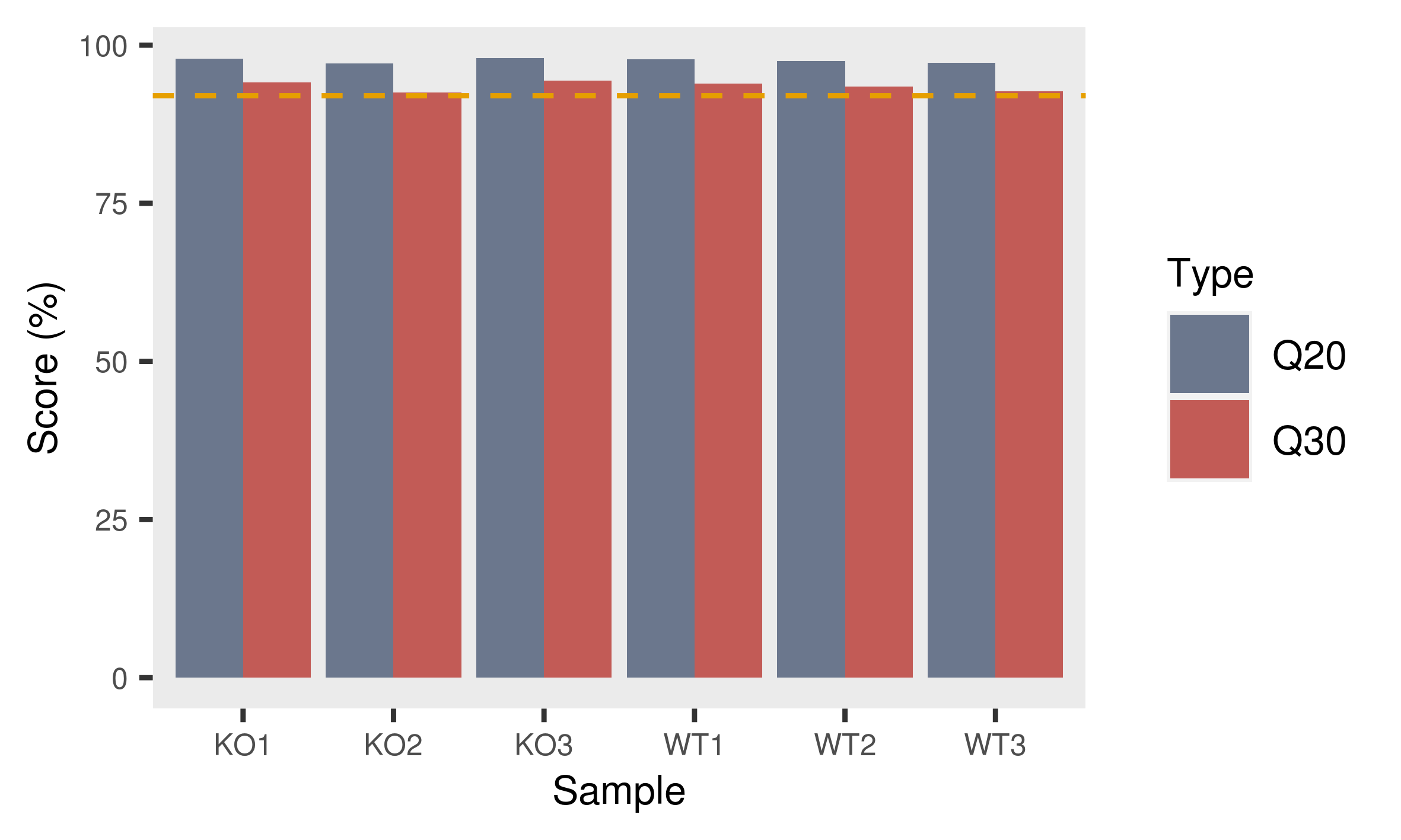 |
| --- | --- |
| (**a**) | (**b**) |

**Figure S4.** Quality control of sequencing data and comparison of mRNA expression profiles between two groups. (**a**) Raw reads and clean reads obtained from sequencing per sample, respectively; (**b**) Q20 and Q30 of each samples. Note: The orange dotted line correspond to 22 reads(M) and 92 score(%) in (a) and (b), respectively**.**

| 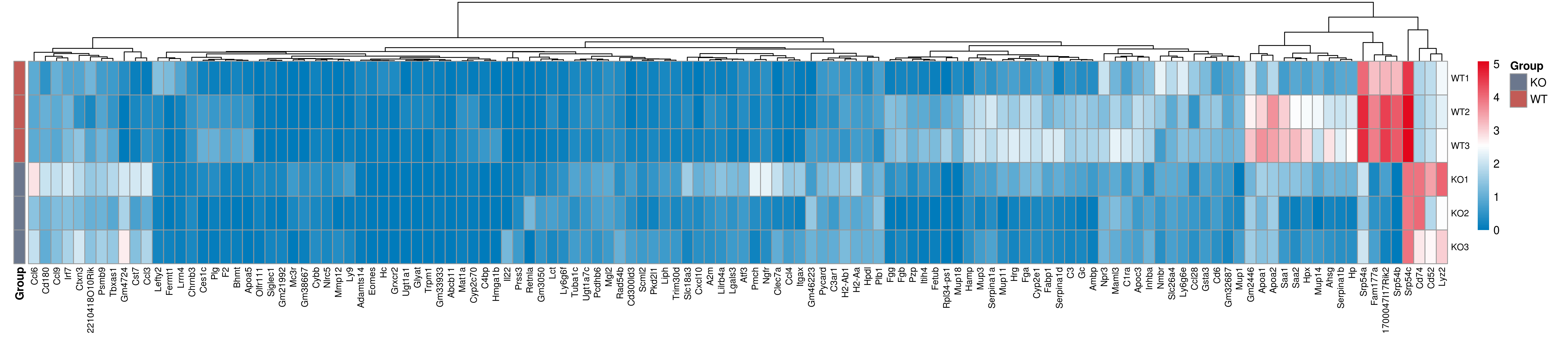 |
| --- |

**Figure S5:** The heatmap to visually assess the results of clustering on the DEGs expression profile. The vertical axis is the log2 of sample (FPKM + 1), and the horizontal axis is the gene. Under the default color matching, the warmer the color block is, the higher the expression level is, and the colder the color block is, the lower the expression level is.

**References**

1. Chen, Y.; Chen, Y.; Shi, C.; Huang, Z.; Zhang, Y.; Li, S.; Li, Y.; Ye, J.; Yu, C.; Li, Z. SOAPnuke: a MapReduce acceleration-supported software for integrated quality control and preprocessing of high-throughput sequencing data. *Gigascience* **2018**, *7*, gix120.

2. Langmead, B.; Salzberg, S.L. Fast gapped-read alignment with Bowtie 2. *Nature methods* **2012**, *9*, 357.

3. Li, B.; Dewey, C.N. RSEM: accurate transcript quantification from RNA-Seq data with or without a reference genome. *BMC bioinformatics* **2011**, *12*, 1-16.
