# Supplementary figures and images for: Behavioral and Gene Expression Analysis of Stxbp6-Knockout Mice
Source: Brain Sci. 2021 Mar 29;11(4):436. doi: 10.3390/brainsci11040436 (PMC8066043; doi:10.3390/brainsci11040436)

**a**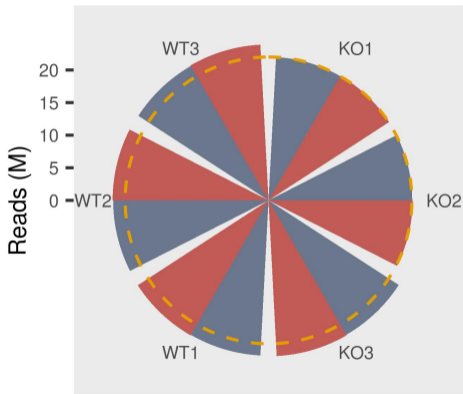**Type**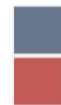

Total clean reads (M)

Total raw reads (M)

**b**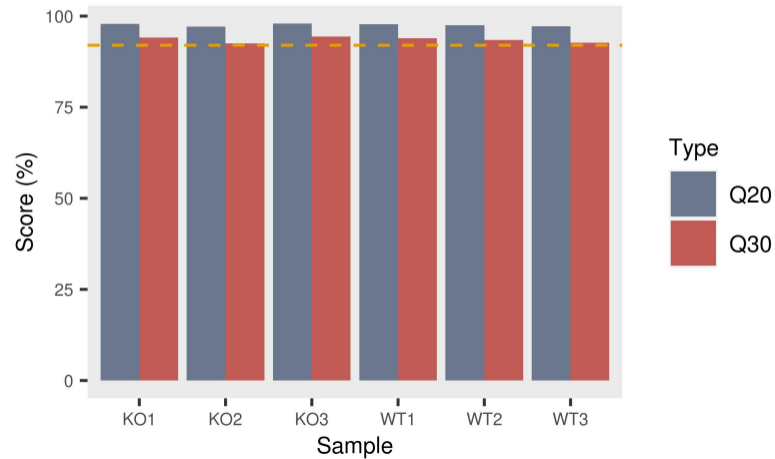**Type**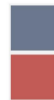

Q20

Q30

Supplement: Supplementary file 1 [file brainsci-11-00436-s001.zip › FigureS4.pdf]

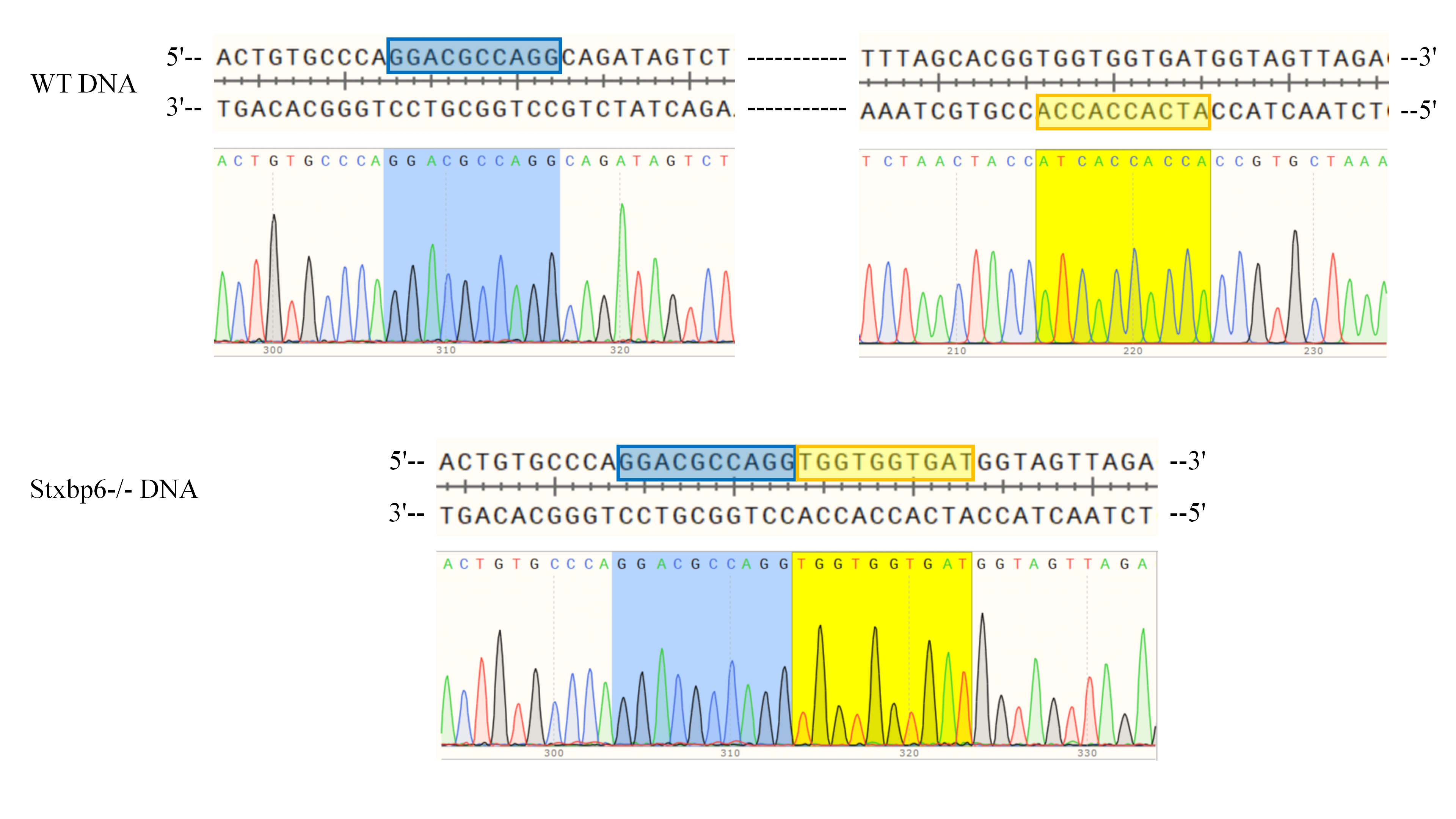

Supplement: Supplementary file 1 [file brainsci-11-00436-s001.zip › Figure_S2.png]

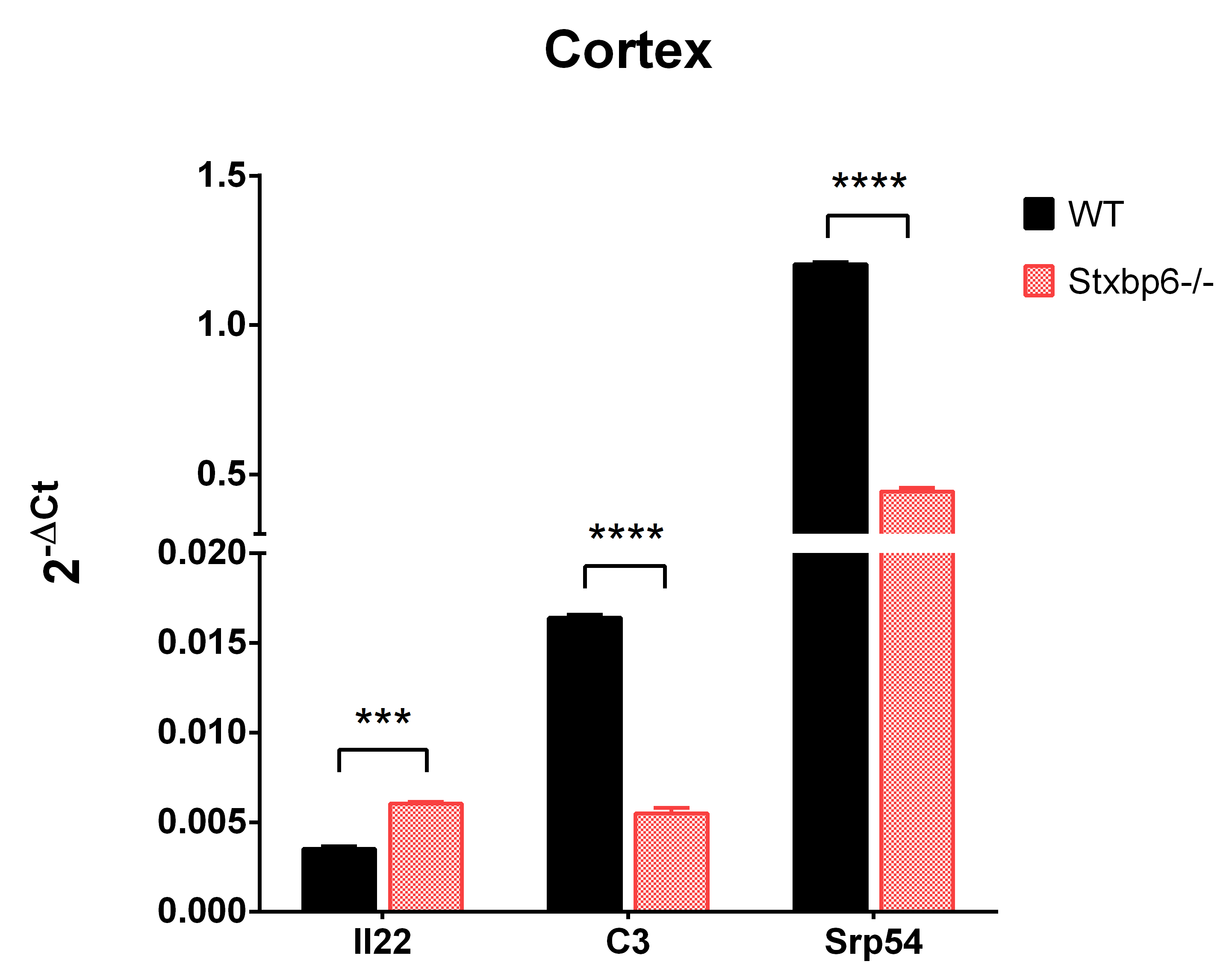

Supplement: Supplementary file 1 [file brainsci-11-00436-s001.zip › Figure_S3_qrtpcr.png]

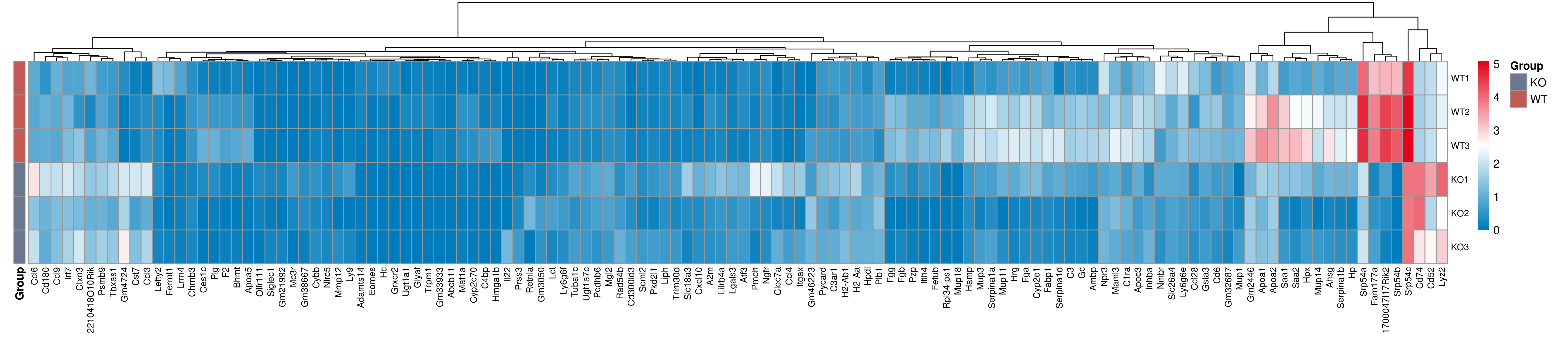

Supplement: Supplementary file 1 [file brainsci-11-00436-s001.zip › Figure_S5_deg_heatmap.png]
